# (1*R*,2*R*)-*N*,*N*′-Dimethyl­cyclo­hexane-1,2-diamine

**DOI:** 10.1107/S1600536808006119

**Published:** 2008-03-12

**Authors:** Carsten Strohmann, Viktoria H. Gessner, Alexander Damme, Stephan Koller, Christian Däschlein

**Affiliations:** a Institut für Anorganische Chemie, Universität Würzburg, Am Hubland, 97074 Würzburg, Germany

## Abstract

The molecule of the title compound, C_8_H_18_N_2_, possesses *C*_2_ symmetry. Owing to its stereochemistry, it is used in the synthesis of chiral ligands and metal complexes for asymmetric synthesis. The cyclo­hexane ring shows a chair conformation with the amino groups in equatorial positions. Contrary to the literature, the title compound is not a liquid, but a crystalline solid at room temperature (293 K). The absolute configuration is assigned from the synthesis.

## Related literature

The synthesis of the title compound is described by Kizirian *et al.* (2005 ▶). For related literature, see: Larrox and Jacobsen (1994 ▶); Cole *et al.* (2005 ▶); Seebach *et al.* (1977 ▶); Strohmann & Gessner (2007 ▶); Strohmann *et al.* (2003 ▶, 2004 ▶); Strohmann, Däschlein & Auer (2006 ▶); Strohmann, Dilsky & Strohfeldt (2006 ▶); Strohmmann & Gessner (2007*a* ▶,*b* ▶).
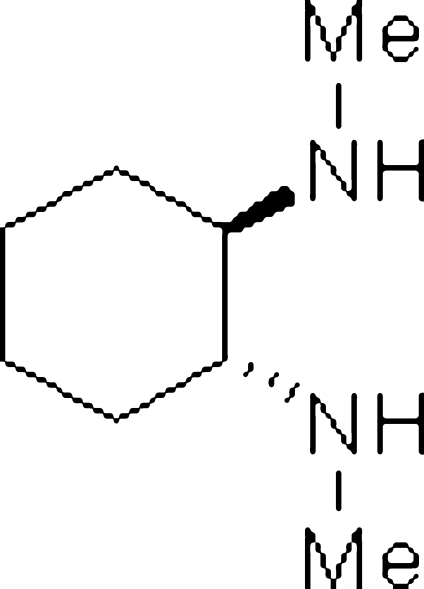


## Experimental

### 

#### Crystal data

C_8_H_18_N_2_
*M* *_r_* = 142.24
Orthorhombic, 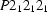

*a* = 7.552 (4) Å
*b* = 8.521 (5) Å
*c* = 14.142 (8) Å
*V* = 910.0 (8) Å^3^
*Z* = 4
Mo *K*α radiation
μ = 0.06 mm^−1^
*T* = 173 (2) K
0.40 × 0.10 × 0.10 mm

#### Data collection

Bruker APEX CCD diffractometer
Absorption correction: multi-scan (*SADABS*; Bruker, 1999 ▶) *T*_min_ = 0.912, *T*_max_ = 0.982
4816 measured reflections
953 independent reflections
784 reflections with *I* > 2σ(*I*)
*R*_int_ = 0.050

#### Refinement

*R*[*F* ^2^ > 2σ(*F* ^2^)] = 0.051
*wR*(*F* ^2^) = 0.111
*S* = 1.08
953 reflections
101 parameters
H atoms treated by a mixture of independent and constrained refinement
Δρ_max_ = 0.12 e Å^−3^
Δρ_min_ = −0.12 e Å^−3^

### 

Data collection: *SMART* (Bruker, 2001 ▶); cell refinement: *SAINT-Plus* (Bruker, 1999 ▶); data reduction: *SAINT-Plus*; program(s) used to solve structure: *SHELXS97* (Sheldrick, 2008 ▶); program(s) used to refine structure: *SHELXL97* (Sheldrick, 2008 ▶); molecular graphics: *ORTEP-3* (Farrugia, 1999 ▶); software used to prepare material for publication: *SHELXL97*.

## Supplementary Material

Crystal structure: contains datablocks I, global. DOI: 10.1107/S1600536808006119/im2055sup1.cif

Structure factors: contains datablocks I. DOI: 10.1107/S1600536808006119/im2055Isup2.hkl

Additional supplementary materials:  crystallographic information; 3D view; checkCIF report

## Figures and Tables

**Table 1:** Hydrogen-bond geometry (Å, °)

*D*—H⋯*A*	*D*—H	H⋯*A*	*D*⋯*A*	*D*—H⋯*A*
N1—H1*N*⋯N2^i^	0.91 (4)	2.36 (4)	3.250 (4)	166 (3)

Symmetry code: (i) 
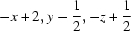
.

**Fig. 1.**
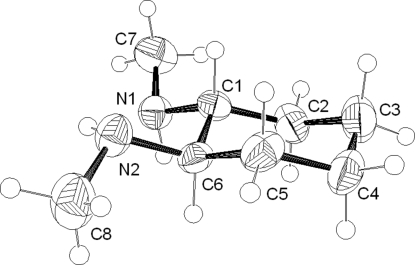
ORTEP plot of the molecular structure of (1R,2R)-N,N'-diemthylcyclohexane-1,2-diamine. Thermal ellipsoids are drawn at the 50% probability level.

**Fig. 2.**
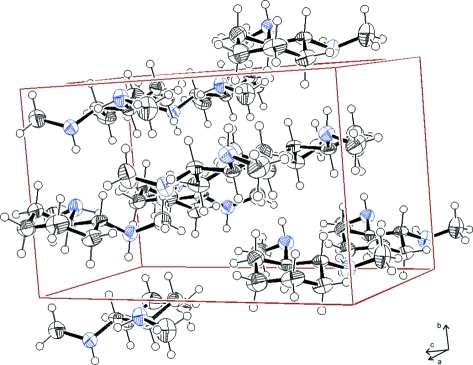
ORTEP plot of the unit cell.

**Fig. 3.**
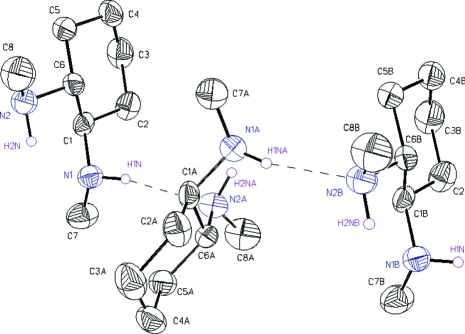
Display of the hydrogen bonding.

## supplementary crystallographic information

### Comment

Due to their strong coordination ability diamine bases have become powerful agents in various fields of chemistry *e.g.* for the deaggregation of organolithium compounds or the coordination of transition metals. Especially chiral amines have attracted special attention in asymmetric synthesis. Thereby, (1*R*,2*R*)-*N,N'*-dimethylcyclohexane-1,2-diamine is an important chiral amine, which serves as a starting material for the synthesis of numerous diamine bases with a cyclohexane framework. The amine crystallizes at room temperature as colourless needles in the orthorhombic crystal system, space group *P*2_1_2_1_2_1_. The asymmetric unit contains one molecule of the *C*_2_ symmetric amine (see figure 1).

In the unit cell molecules are interconnected *via* hydrogen bonding to give infinite layers (see figure 2). H atoms (H1N) are arranged in direction to the nitrogen atom (N2) of an adjacent molecule (N1—HN1—N2' angle: 166 (3)°). However, the long N1—N2' distance of 3.250 (4) Å and the short N1—HN1 distance of 0.91 (4) Å indicate weak N–H···N hydrogen bonds.

### Experimental

Treatment of the enantiomerically pure (*R,R*)-1,2-diammoniumcyclohexane mono-(+)-tartrate with two equivalents of ethylchloroformate in the presence of a stochiometric amount of NaOH resulted in the formation of diethyl-(1*R*,2*R*)-cyclohexane-1,2-diyldicarbamat. Subsequent reduction with an excess of LiAlH_4_ gave colourless crystals of the title compound during bulb-to-bulb destillation. Contrary to a formerly published synthesis, (1*R*,2*R*)-*N*,*N*'-diemthylcyclohexane-1,2-diamine is not liquid but a highly hygroscopic crystalline solid.

^1^H-NMR (500.1 MHz, CDCl_3_): 0.86–0.94 (m, 2H; C*H_2_*CHN), 1.13–1.19 (m, 2H; C*H_2_*CH_2_CHN), 1.61–1.67 (m, 2H; C*H_2_*CH_2_CHN), 1.68–1.75 (br, 2H, N*H*), 1.93–2.00 (m, 2H; C*H_2_*CHN), 2.02–2.06 (m, 2H; C*H*NCHN), 2.33 (s, 6H; NC*H_3_*).

^13^C-NMR (100.6 MHz, CDCl_3_): 25.0 (*C*H_2_CH_2_CHN), 30.8 (*C*H_2_CHN), 33.7 (*C*H_3_), 63.2 (*C*HN).

### Refinement

Refinement was accomplished by full-matrix least-squares methods (based on *F_o_*^2^, *SHELXL97*); anisotropic thermal parameters for all non-H atoms in the final cycles; the H atoms were refined on a riding model in their ideal geometric positions, except for H(1 N) and H(2 N), which were refined independently.

### Crystal data

**Table:**

C_8_H_18_N_2_	*F*_000_ = 320
*M**_r_* = 142.24	*D*_x_ = 1.038 Mg m^−^^3^
Orthorhombic, *P*2_1_2_1_2_1_	Melting point: 313 K
Hall symbol: P 2ac 2ab	Mo *K*α radiation λ = 0.71073 Å
*a* = 7.552 (4) Å	θ = 2.8–25.0º
*b* = 8.521 (5) Å	µ = 0.06 mm^−^^1^
*c* = 14.142 (8) Å	*T* = 173 (2) K
*V* = 910.0 (8) Å^3^	Needle, colourless
*Z* = 4	0.40 × 0.10 × 0.10 mm

### Data collection

**Table:**

Bruker APEXCCD diffractometer	953 independent reflections
Radiation source: fine-focus sealed tube	784 reflections with *I* > 2σ(*I*)
Monochromator: graphite	*R*_int_ = 0.050
*T* = 173(2) K	θ_max_ = 25.0º
ω scans	θ_min_ = 2.8º
Absorption correction: multi-scan(SADABS; Bruker, 1999)	*h* = −8→8
*T*_min_ = 0.912, *T*_max_ = 0.982	*k* = −10→9
4816 measured reflections	*l* = −16→16

### Refinement

**Table:**

Refinement on *F*^2^	Secondary atom site location: difference Fourier map
Least-squares matrix: full	Hydrogen site location: inferred from neighbouring sites
*R*[*F*^2^ > 2σ(*F*^2^)] = 0.051	H atoms treated by a mixture of independent and constrained refinement
*wR*(*F*^2^) = 0.111	*w* = 1/[σ^2^(*F*_o_^2^) + (0.0405*P*)^2^ + 0.258*P*] where *P* = (*F*_o_^2^ + 2*F*_c_^2^)/3
*S* = 1.08	(Δ/σ)_max_ < 0.001
953 reflections	Δρ_max_ = 0.12 e Å^−^^3^
101 parameters	Δρ_min_ = −0.11 e Å^−^^3^
Primary atom site location: structure-invariant direct methods	Extinction correction: none

### Special details

**Table:**

Geometry. All e.s.d.'s (except the e.s.d. in the dihedral angle between two l.s. planes) are estimated using the full covariance matrix. The cell e.s.d.'s are taken into account individually in the estimation of e.s.d.'s in distances, angles and torsion angles; correlations between e.s.d.'s in cell parameters are only used when they are defined by crystal symmetry. An approximate (isotropic) treatment of cell e.s.d.'s is used for estimating e.s.d.'s involving l.s. planes.
Refinement. Refinement of *F*^2^ against ALL reflections. The weighted *R*-factor *wR* and goodness of fit *S* are based on *F*^2^, conventional *R*-factors *R* are based on *F*, with *F* set to zero for negative *F*^2^. The threshold expression of *F*^2^ > σ(*F*^2^) is used only for calculating *R*-factors(gt) *etc*. and is not relevant to the choice of reflections for refinement. *R*-factors based on *F*^2^ are statistically about twice as large as those based on *F*, and *R*- factors based on ALL data will be even larger.

### Fractional atomic coordinates and isotropic or equivalent isotropic displacement parameters (Å^2^)

**Table:**

	*x*	*y*	*z*	*U*_iso_*/*U*_eq_	
C1	0.8581 (4)	0.3857 (3)	0.28989 (19)	0.0346 (7)	
H1	0.8702	0.5000	0.3046	0.042*	
C2	0.6814 (4)	0.3316 (4)	0.3294 (2)	0.0477 (9)	
H2A	0.6785	0.3525	0.3982	0.057*	
H2B	0.6703	0.2168	0.3202	0.057*	
C3	0.5242 (4)	0.4129 (5)	0.2827 (3)	0.0608 (11)	
H3A	0.4128	0.3663	0.3067	0.073*	
H3B	0.5247	0.5256	0.2999	0.073*	
C4	0.5304 (4)	0.3970 (4)	0.1764 (3)	0.0528 (10)	
H4A	0.5131	0.2857	0.1586	0.063*	
H4B	0.4333	0.4590	0.1479	0.063*	
C5	0.7059 (4)	0.4540 (4)	0.1385 (2)	0.0458 (9)	
H5A	0.7174	0.5679	0.1509	0.055*	
H5B	0.7091	0.4381	0.0691	0.055*	
C6	0.8605 (4)	0.3685 (3)	0.18321 (18)	0.0331 (7)	
H6	0.8514	0.2545	0.1671	0.040*	
C7	1.0546 (5)	0.3573 (4)	0.4252 (2)	0.0581 (10)	
H7A	1.0807	0.4699	0.4250	0.087*	
H7B	1.1583	0.2994	0.4480	0.087*	
H7C	0.9537	0.3367	0.4670	0.087*	
C8	1.0664 (5)	0.3767 (5)	0.0508 (2)	0.0644 (11)	
H8A	1.0492	0.2629	0.0465	0.097*	
H8B	1.1894	0.4025	0.0350	0.097*	
H8C	0.9866	0.4296	0.0065	0.097*	
N1	1.0117 (3)	0.3064 (4)	0.32977 (18)	0.0389 (7)	
H1N	0.985 (4)	0.203 (4)	0.329 (2)	0.056 (10)*	
N2	1.0282 (4)	0.4287 (3)	0.14644 (19)	0.0413 (7)	
H2N	1.109 (4)	0.391 (4)	0.193 (2)	0.045 (9)*	

### Atomic displacement parameters (Å^2^)

**Table:**

	*U*^11^	*U*^22^	*U*^33^	*U*^12^	*U*^13^	*U*^23^
C1	0.0385 (17)	0.0216 (15)	0.0438 (17)	0.0043 (16)	0.0006 (14)	0.0013 (13)
C2	0.044 (2)	0.0420 (19)	0.057 (2)	0.0050 (17)	0.0110 (17)	0.0051 (17)
C3	0.039 (2)	0.053 (2)	0.090 (3)	0.0029 (19)	0.010 (2)	0.007 (2)
C4	0.0335 (19)	0.0403 (19)	0.085 (3)	−0.0023 (17)	−0.0126 (19)	0.0081 (19)
C5	0.046 (2)	0.0355 (19)	0.056 (2)	−0.0032 (17)	−0.0131 (16)	0.0054 (16)
C6	0.0341 (16)	0.0251 (16)	0.0400 (17)	−0.0025 (15)	−0.0054 (14)	−0.0008 (13)
C7	0.068 (2)	0.055 (2)	0.052 (2)	0.010 (2)	−0.0109 (18)	−0.0046 (18)
C8	0.057 (2)	0.085 (3)	0.052 (2)	−0.008 (2)	0.0120 (18)	0.005 (2)
N1	0.0389 (15)	0.0388 (16)	0.0390 (15)	0.0029 (14)	−0.0033 (13)	0.0002 (13)
N2	0.0370 (16)	0.0514 (18)	0.0355 (15)	−0.0048 (14)	0.0004 (13)	0.0048 (13)

### Geometric parameters (Å, °)

**Table:**

C1—N1	1.455 (4)	C5—H5A	0.9900
C1—C6	1.516 (4)	C5—H5B	0.9900
C1—C2	1.519 (4)	C6—N2	1.462 (4)
C1—H1	1.0000	C6—H6	1.0000
C2—C3	1.525 (4)	C7—N1	1.454 (4)
C2—H2A	0.9900	C7—H7A	0.9800
C2—H2B	0.9900	C7—H7B	0.9800
C3—C4	1.511 (5)	C7—H7C	0.9800
C3—H3A	0.9900	C8—N2	1.452 (4)
C3—H3B	0.9900	C8—H8A	0.9800
C4—C5	1.510 (4)	C8—H8B	0.9800
C4—H4A	0.9900	C8—H8C	0.9800
C4—H4B	0.9900	N1—H1N	0.91 (4)
C5—C6	1.515 (4)	N2—H2N	0.96 (3)
			
N1—C1—C6	109.4 (2)	C4—C5—H5B	109.2
N1—C1—C2	114.6 (2)	C6—C5—H5B	109.2
C6—C1—C2	110.3 (3)	H5A—C5—H5B	107.9
N1—C1—H1	107.4	N2—C6—C5	110.5 (2)
C6—C1—H1	107.4	N2—C6—C1	109.3 (2)
C2—C1—H1	107.4	C5—C6—C1	111.1 (3)
C1—C2—C3	112.8 (3)	N2—C6—H6	108.6
C1—C2—H2A	109.0	C5—C6—H6	108.6
C3—C2—H2A	109.0	C1—C6—H6	108.6
C1—C2—H2B	109.0	N1—C7—H7A	109.5
C3—C2—H2B	109.0	N1—C7—H7B	109.5
H2A—C2—H2B	107.8	H7A—C7—H7B	109.5
C4—C3—C2	111.4 (3)	N1—C7—H7C	109.5
C4—C3—H3A	109.3	H7A—C7—H7C	109.5
C2—C3—H3A	109.3	H7B—C7—H7C	109.5
C4—C3—H3B	109.3	N2—C8—H8A	109.5
C2—C3—H3B	109.3	N2—C8—H8B	109.5
H3A—C3—H3B	108.0	H8A—C8—H8B	109.5
C5—C4—C3	110.6 (3)	N2—C8—H8C	109.5
C5—C4—H4A	109.5	H8A—C8—H8C	109.5
C3—C4—H4A	109.5	H8B—C8—H8C	109.5
C5—C4—H4B	109.5	C7—N1—C1	113.5 (2)
C3—C4—H4B	109.5	C7—N1—H1N	111 (2)
H4A—C4—H4B	108.1	C1—N1—H1N	106 (2)
C4—C5—C6	111.9 (3)	C8—N2—C6	113.4 (3)
C4—C5—H5A	109.2	C8—N2—H2N	114.6 (19)
C6—C5—H5A	109.2	C6—N2—H2N	100.9 (19)

### Hydrogen-bond geometry (Å, °)

**Table:**

*D*—H···*A*	*D*—H	H···*A*	*D*···*A*	*D*—H···*A*
N1—H1N···N2^i^	0.91 (4)	2.36 (4)	3.250 (4)	166 (3)

Symmetry codes: (i) −*x*+2, *y*−1/2, −*z*+1/2.
